# Rosmarinic acid mitigates stress-induced gastric ulcers by targeting the ACE2/GPX4 interaction

**DOI:** 10.3389/fphar.2026.1891305

**Published:** 2026-08-24

**Authors:** Tao Zeng, Feng Li, Fei-yang Wei, Zhi-qiang Zheng, Jing-wen Zheng, Tai-fu Luo, Jing-hong Gao, Ruo-ting Men, Zhi-hui Yi, Li-hong Wan, Ling Zhu

**Affiliations:** 1 Department of Pharmacology, West China School of Basic Medical Sciences & Forensic Medicine, Sichuan University, Chengdu, Sichuan, China; 2 Department of Gastroenterology, 363 Hospital, Chengdu, Sichuan, China; 3 Department of Gastroenterology, West China Hospital, Sichuan University, Chengdu, Sichuan, China

**Keywords:** ACE2, ferroptosis, GPx4, rosmarinic acid, water restraint-induced gastric ulcer mouse model

## Abstract

**Introduction:**

The renin-angiotensin system (RAS), a pivotal target in gastric ulcers, has been shown to regulate ferroptosis in bronchial epithelial cells. Rosmarinic acid (RA) possesses significant anti-ulcer and anti-ferroptosis effects. However, it is still unclear whether RA alleviates gastric ulcers by preventing ferroptosis.

**Methods:**

We used a water restraint-induced ulcer mouse model, and an LPS-induced GEC-1 cells inflammation model to observe stress-induced damage both *in vivo* and *in vitro*, and to explore the protective effects and mechanisms of RA on such damage.

**Results:**

We identified 14 DEGs related to ferroptosis between chronic gastritis and gastric ulcer. Some form a PPI network with RAS-related protein and serum ACE2 was significantly increased in ulcers. Molecular docking and molecular dynamics simulations revealed stable binding between GPX4 and ACE2, and the incorporation of RA markedly boosts its stability. Also, RA administration significantly mitigated SGU via upregulation of GPX4 and ACE2 in cold water-immersion restraint stress (CWIR) mice.

**Discussion:**

Our research demonstrates that RA may be a therapeutic strategy to improve SGU by targeting the ACE2/GPX4 interaction.

## 1 Introduction

Stress is one of the notable inducers of gastric ulcers, which are associated with a high mortality rate (Gyires and Feher, 2017; Krag M et al., 2015). Under stress conditions, an acute gastric mucosal lesion occurs, characterized by increased gastric acid secretion, the accumulation of inflammatory cells, changes in blood flow, and the reduction in the protective mucus barrier (Magierowski et al., 2017). Currently, treatments for stress-induced gastric ulcers (SGU) encompass prevention and pharmacological intervention, especially H2 receptor antagonists or proton pump inhibitors (Barbateskovic et al., 2019)^.^ However, the therapeutic outcomes are not always satisfactory owing to the side effects from prolonged treatment, including elevated gastric pH, gastric acidemia, and an increased risk of *Clostridium difficile* diarrhea, thrombocytopenia, and interstitial nephritis (Koyyada, 2021). Therefore, clarifying the pathogenesis of SGU and identifying novel, specific targets for diagnosis and treatment holds immense significance.

Ferroptosis is a form of programmed cell death characterized by mitochondrial damage, induced by lipid peroxidation and an excess of iron accumulation (Stockwell et al., 2017). Recently, growing evidence highlights the critical role of ferroptosis in the pathogenesis of alcohol-induced GU (Meng et al., 2024; Zhang et al., 2023). Additionally, the suppression of angiotensin-converting enzyme (ACE) (Brzozowski et al., 2017), or activation of angiotensin (Ang) 2 converting enzyme (ACE2) pathway (Souza et al., 2016), emerges as a crucial therapeutic target in the management of gastric ulcers, as it augments gastric microvascular perfusion and enhances gastric tissue oxygenation (Truse et al., 2019). Importantly, our previous study has demonstrated that the imbalance between ACE and ACE2 plays a crucial role in endotoxin-induced ferroptosis of bronchial epithelial cells (Zeng et al., 2024), implying the potential role for renin-angiotensin system (RAS)-mediated ferroptosis in the pathogenesis of SGU.

Rosmarinic acid (RA) is a hydrophilic polyphenol that is ubiquitous in numerous traditional Chinese medicinal herbs, including *Rosemary, Perilla frutescens L.,* and *Prunella* (Li et al., 2025). As a powerful agent with direct free radical scavenging ability, it possesses a variety of biological activities, including inhibiting endoplasmic reticulum stress (ERS) (Li et al., 2025), antioxidant activity (Liang et al., 2026), anti-apoptotic, and anti-inflammatory (Noor et al., 2022). Recently, increasing studies have highlighted the significance of RA in the treatment of various gastric ulcer models (Kangwan et al., 2019; do Nascimento et al., 2020; Wang et al., 2021). Also, our previous research has verified that RA not only serves as an ACE inhibitor but also reestablishes the ACE/ACE2 balance, thereby inhibiting RAS-mediated ferroptosis in bronchial epithelial cells (Zeng et al., 2024).

In this study, we hypothesize that RA mitigates SGU via modulating RAS-induced ferroptosis in gastric epithelial cells. To validate this hypothesis, firstly we reveal the role of RAS-mediated ferroptosis in gastric ulcer occurrence based on the transcriptome data of clinical gastric mucosal tissues and bioinformatics analysis. Next, we established the LPS-induced gastric epithelial cell inflammation model and cold water-immersion restraint stress (CWIR) mice model to evaluate the protective effect of RA on SGU. Finally, the molecular mechanism of RA mitigating SGU was elucidated by molecular docking and molecular dynamics simulation.

## 2 Materials and methods

### 2.1 Human samples RNA sequencing (RNA-seq) and bioinformatics analysis

The study was conducted in accordance with the Basic and Clinical Pharmacology and Toxicology policy for experimental and clinical studies (Tveden-Nyborg et al., 2023). From 1 September 2024, to 1 November 2024, we collected gastric mucosa samples from endoscopic resections at the 363 Hospital from two distinct groups: chronic gastritis patients and gastric ulcer patients. Baseline characteristics are shown in Table 1 and the patients with other underlying conditions or infections were excluded. These protocols were approved by the ethics committee of the West China Hospital of Sichuan University, and 363 Hospital (No: 2024–862).

**TABLE 1:** Clinical characteristics of patients (n = 6).

Variables	Chronic gastritis group	Gastric ulcer group	P-value
Age (years)	49 ± 6.81	41.5 ± 10.07	>0.05
Gender (male: female)	4:2	4:2	>0.05
Previous treatment	No	No	>0.05

The gastric mucosa tissues were sent to Hangzhou Kaitai Biotechnology Co., Ltd. for transcriptome sequencing. Samples that passed quality control were sequenced on the DNBSEQ platform. The clean data were obtained through filtering using SOAPnuke (v1.5.6), then aligned to the reference genome using HISAT2 (v2.1.0) software and the reference gene set using Bowtie2 (v2.3.4.3). Gene expression quantification was performed using RSEM (v1.3.1) software, and differential gene expression analysis was conducted using DESeq2 (v1.4.5) with a cutoff of Q value ≤0.05.

Gene ontology (GO) and Kyoto Encyclopedia of Genes and Genomes (KEGG) enrichment analysis of differentially expressed genes (DEGs) was conducted using the DAVID platform (https://david-d.ncifcrf.gov/), with a Q value ≤0.05 as the threshold, which is defined as significant enrichment in candidate genes that meet this condition. The analysis results were visualized using the R language (4.4.3 version).

DEGs from RNA-seq were intersected with ferroptosis-related genes (FRGs) and visualized with Venn 2.1 (https://bioinfogp.cnb.csic.es/tools/venny/index.html). The Retrieval of Interacting Genes/Proteins (STRING) version 12.0 database (https://cn.string-db.org/) was used to perform the Protein-protein interaction (PPI) network analysis.

### 2.2 ACE2 measurement

Blood samples of patients were collected and centrifuged. The serum was collected to determine the concentration of ACE2 (Cloud-Clone, Corp. China) using an enzyme-linked immunosorbent assay (ELISA) according to the manufacturer’s instructions.

### 2.3 Single-cell sequencing dataset analysis

A database of Deeply Integrated Single-Cell Omics data of human (DISCO, https://disco.bii.a-star.edu.sg/) was used to analyze the cell-type-specific expression of ACE2 in the stomach tissue.

### 2.4 Drug sources

RA for mice administration was purchased from Macklin (R817282-1g, HPLC ≥98%, China). RA for the *in vitro* experiment was purchased from Yuanye (B20862, HPLC ≥98%, China). Esomeprazole Magnesium Enteric-coated Capsules (H20213071, 20 mg) were obtained from Chia Tai Tianqing Pharmaceutical Group Co., Ltd, China. Lipopolysaccharide (LPS) was obtained from Sigma-Aldrich (L6529, USA). Captopril was purchased from Yuanye (S30916, HPLC ≥98%, China). Ferrostatin-1 was purchased from Harvey Bio (FL0785, HPLC ≥99%, China).

### 2.5 Protein-protein molecular docking and molecular dynamics simulation

GRAMM (http://gramm.compbio.ku.edu/) was used to rigidly dock the ACE2 protein with SLC7A11 or GPX4 protein. Groningen Machine for Chemicals Simulations (GROMACS) v2022.03 with AMBER99SB-ILDN, and Three-point transferable intermolecular potential (TIP3P) water model, and the lowest binding energy came from molecular docking was selected for performing MD verification. The protein-ligand complex was simulated for 100 ns. The Xmgrace software was used to analyze the root mean square deviation (RMSD) and root mean square fluctuation (RMSF). The molecular mechanics/Poisson-Boltzmann surface area (MM/PBSA) was used to calculate the binding free energy.

### 2.6 RA-protein complex molecular docking and molecular dynamics simulation

AutoDock Vina v1.2.3 software was used for molecular docking analysis for the ACE2-GPX4 complex and rosmarinic acid. GROMACS v2022.03 performed a 100 ns molecular dynamics simulation on the protein-small molecule complexes obtained from molecular docking with AMBER99SB-ILDN as the force field.

### 2.7 Cell culture and treatment

Human gastric epithelial GEC-1 cells were donated by Professor Wang Bao-ning and cultured in DMEM medium supplemented with 10% fetal bovine serum (FBS) and 1% penicillin-streptomycin. The cells were maintained in a humidified incubator at 37 °C with 5% CO_2_. Next, cells were plated at 1 × 10^6^ cells per well in six-well plates. Twenty-four hours later, GEC-1 cells were incubated with LPS (15 μg/mL) for 24 h. All experiments were conducted in triplicate and repeated three times. Captopril (20 µM) or Ferrostatin-1 (1 µM) was added to the medium 6 h after LPS stimulation.

### 2.8 Cold water-immersion restraint stress (CWIR) animal model and allocations

According to the Animal ethics and experimental procedures approved by West China Hospital of Sichuan University and the guidelines of the China Council on Animal Care (No. 20240308066), 5-week-old specific pathogen-free (SPF) Kunming mice (weight of 18–22 g) acquired from Da-Shuo Biological Technology Co., Ltd (Chengdu, China) were acclimatization (Temperature: 22 °C–24 °C, relative humidity: 50%–60%, and a 12-h light/dark cycle) for 5 days. Then, all mice were randomly divided into five groups: control group, model group, RA 4 mg/kg group, RA 20 mg/kg group, and esomeprazole group (3.03 mg/kg), with 6 in each group (3 male and 3 female) (Yi et al., 2026). The doses of Rosmarinic Acid were selected based on the literature and our preliminary experiment (Kangwan et al., 2019; Wang et al., 2021; Zeng et al., 2024). All mice were pre-treated with the corresponding drugs via the designated routes and doses for 4 days, while the control group and model group were given an equal dose of saline for 4 days. After a 24-h fast and a 4-h no-water fast, mice were restrained in polyvinyl chloride tubes and vertically immersed in cold water with a temperature of around 15 °C–20 °C for 6 h, at the level of the sternum xiphoid. Meanwhile, the mice in the control group were placed in the cages under the same external environment. Next, all the mice were treated with the corresponding drugs for another 4 days and euthanized via intraperitoneal injection of sodium amobarbital. The agent was diluted with normal saline to 10 mg/mL and administered at 40 mg/kg according to the body weight of mice. Gastric tissues were rapidly collected for subsequent experiments.

### 2.9 Assessment of gastric tissue injury

The gastric cavity of the mice was opened along the greater curvature and rinsed completely with 5 mL of distilled water. Then, the stomachs were blotted dry with filter paper, spread out, and photographed. The lesion length in the flattened stomach samples was measured using a vernier caliper. The gastric ulcer index (GUI) was calculated as follows: under the dissecting microscope, the amount of bleeding, the size of the ulcers, and their distribution on the gastric mucosa were observed. The longest diameter of each ulcer was measured. Ulcers with the longest diameter ≤1 mm were scored as 1 point; those >1–2 mm were scored as 2 points; those >2–3 mm were scored as 3 points; those >3–4 mm were scored as 4 points; and those >4 mm were scored as 5 points (Liu et al., 2021).

### 2.10 Detection of pepsin activity

Approximately 0.1 g of gastric tissue was weighed and homogenized in 1 mL of extraction buffer from the Pepsin Activity Assay Kit (Beijing Solarbio Science and Technology Co., Ltd., Cat. No. 20210125) at 4 °C. The homogenate was centrifuged at 10,000 rpm for 10 min, and the supernatant was collected. The protein concentration in the supernatant was determined using the BCA Protein Assay Kit at 562 nm. Pepsin activity was measured using the UV spectrophotometry method with the Pepsin Activity Assay Kit. The absorbance of all samples was measured at 275 nm, and the pepsin activity (U•mg prot^-1^) was calculated using the formula: Pepsin Activity (U•mg prot^-1^) = 1.31 × (A_sample_ - A_blank_)/Cpr.

### 2.11 Hematoxylin and eosin (HE) staining and PAS

The gastric tissues were fixed in 4% paraformaldehyde at 4 °C for 24 h, embedded in paraffin, and sectioned at a thickness of 4 μm. The sections were stained with HE to observe the pathological morphological changes in the gastric mucosa and stained with periodic acid-Schiff (PAS). The morphological features of gastric tissue injury, assessed through double-blind observation utilizing a light microscope (DM250, LEICA, Germany), were categorized on a scale ranging from 0 (normal) to 4 (severe). Scores were allocated separately for ulcer depth, bleeding, and inflammation, and the total gastric injury scores were obtained by adding the individual scores together (Liu et al., 2021; Yi et al., 2026). PAS staining was used to assess the proliferation of goblet cells. For quantitative analysis of mucus production, digital images were acquired under constant exposure parameters, and the ratio of PAS-positive stained area to total epithelial area was determined using ImageJ software.

### 2.12 Immunohistochemical (IHC) of GPX4

Gastric tissues were collected from mice and fixed in 10% neutral buffered formalin for 24 h. After dehydration and clearing, the tissues were embedded in paraffin and sectioned into 4-μm slices, which were mounted on poly-L-lysine-coated slides. The sections were subjected to antigen retrieval by heating in citrate buffer for 15 min in a microwave. Following cooling, endogenous peroxidase activity was quenched with 3% hydrogen peroxide, and the sections were blocked with 5% normal goat serum for 30 min. Primary antibodies (GPX4 (Abcam, ab125066)) were diluted to 1:100, and the membrane was incubated with them overnight at 4 °C. Secondary antibodies (goat anti-rabbit IgG labeled by HRP) were applied for 1 h at room temperature. Sections were then stained with DAB, counterstained with hematoxylin, dehydrated, and mounted. Finally, the sections were observed under a microscope, photographed, and the average optical density was calculated using image analysis software for quantitative analysis.

### 2.13 Western blot

Gastric tissues were lysed using Western blot lysis buffer to extract proteins. The proteins were separated by 10% SDS-PAGE gel electrophoresis and transferred onto a PVDF membrane for 90 min. The membrane was blocked with 5% skim milk for 2 h. The primary antibodies were incubated overnight at 4 °C. The specific antibodies used were ACE (Abcam, ab183591, 1:1000), ACE2 (Proteintech, 21115-1-AP, 1:1000), GPX4 (Abcam, ab125066, 1:1000), β-actin (Cell Signaling Technology, 3700S, 1:1000), and GAPDH (Cell Signaling Technology, 97166S, 1:1000). The secondary antibodies (anti-rabbit IgG (SAB L3012, 1:20,000)) were incubated for 90 min, and the signals were detected using chemiluminescence. The band densities were analyzed using ImageJ software.

### 2.14 Statistical analysis

The data were analyzed by GraphPad Prism 8.0 software and were expressed as mean ± standard deviation (SD). Continuous and normally distributed variables were expressed as the mean ± SD and analyzed by Student’s t-test or one-way analysis of variance (ANOVA) with Dunnett *post hoc* test. All tests were two-sided. P < 0.05 was considered statistically significant.

## 3 Results

### 3.1 RAS-mediated ferroptosis contributes to gastric ulcer occurrence

**FIGURE 1:**
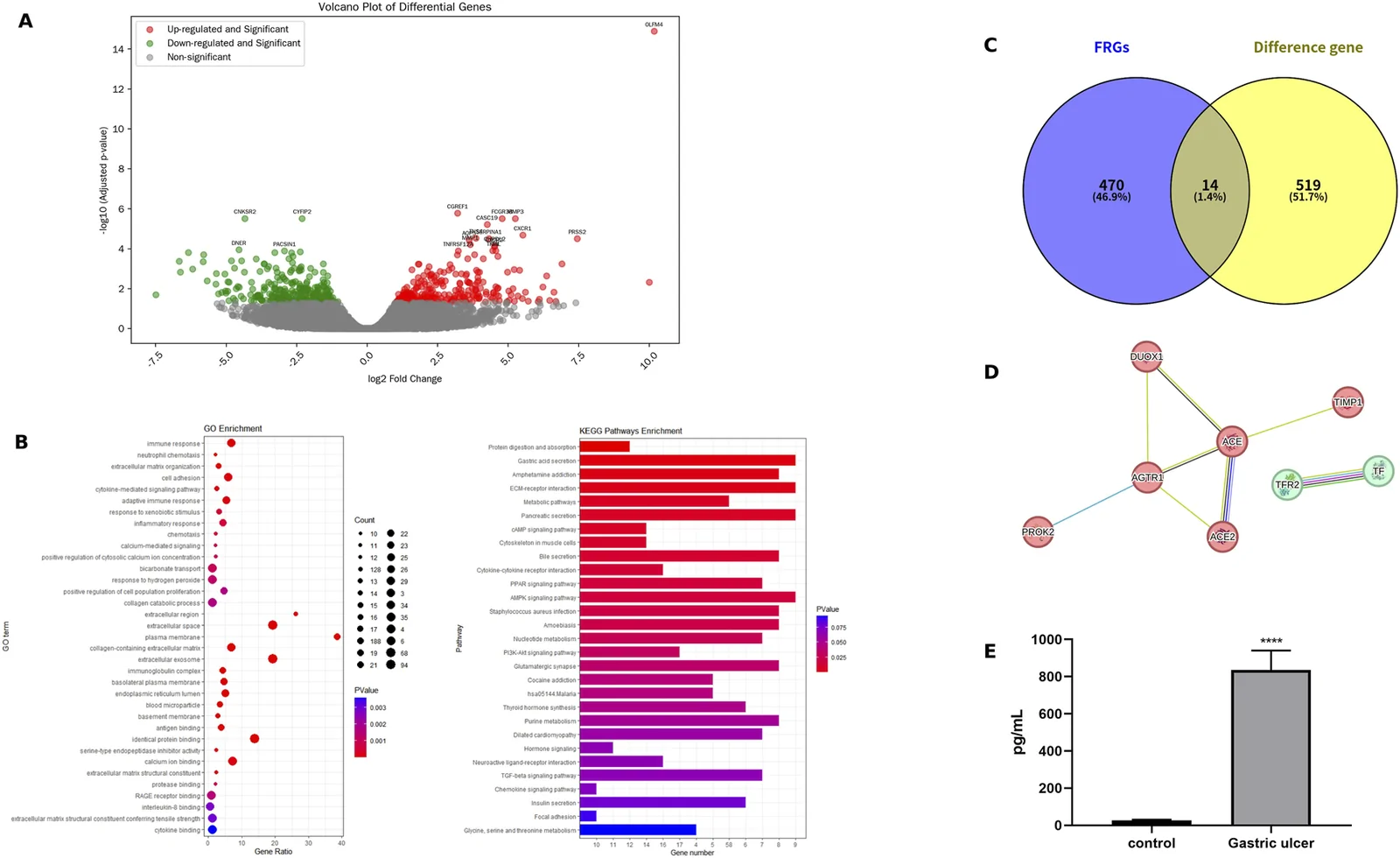
RAS-mediated ferroptosis contributes to gastric ulcer occurrence. **(A)** Volcano plot depicting RNAs that are upregulated (red) and downregulated (green); **(B)** KEGG enrichment analysis; **(C)** Venn diagram showing 14 ferroptosis-related DEGs. The yellow circle represents 533 DEGs between groups, the blue circle represents 484 ferroptosis-related genes, and the overlap represents 14 ferroptosis-related DEGs. **(D)** PPI analysis of the overlapped genes with ACE, ACE2, and AT1R was conducted using STRING. **(E)** Serum ACE2 concentration in patients by ELISA. Data are means ± SD and analyzed by Student’s t-test or one-way analysis of variance (ANOVA) with Dunnett *post hoc* test. All tests were two-sided (n = 6). P < 0.05 was considered statistically significant. *P < 0.05, versus the Ctrl group; #P < 0.05, versus the Model group.

To better understand the difference between chronic gastritis and ulcers, RNA-Seq was used to determine the DEGs of gastric mucosa tissues from chronic gastritis patients and gastric ulcer patients. As shown in Figure 1A, 533 DEGs were screened (Q value <0.05, |log2FC| ≥ 1), including 272 upregulated genes and 261 downregulated genes in ulcer gastric tissues. GO and KEGG pathway enrichment analysis showed that these proteins were enriched in immune responses, cell adhesion, and inflammatory responses, with several pathways, such as the gastric acid secretion, metabolic pathway, and cAMP signaling pathway (Figure 1B). Next, utilizing data from ferroptosis-related genes (FRGs) dataset, a total of 14 overlapping genes related to both DEGs and FRGs were identified (Figure 1C), including DUOX1, TF, TFR2, AQP5, GRIA3, AGPS, ACADSB, SNCA, FADS2, LIFR, TIMP1, FNDC5, ETV4, and PROK2. Given the imbalance of ACE/ACE2 plays a role in promoting ferroptosis (Zeng et al., 2024), STRING was employed to establish the PPI network, thereby elucidating the interactions among PROK2, DUOX1, TIMP1, and RAS-related proteins (Figure 1D). Finally, we measured the level of serum ACE2 in patients’ peripheral blood, and found that ACE2 concentration was significantly increased in the gastric ulcer patients (Figure 1E, P < 0.05), compared with chronic gastritis patients. These results indicate that ACE2 plays a critical role in the occurrence of gastric ulcers.

### 3.2 Epithelial ACE2/GPX4 interaction is a key mechanism of gastric ulcer

**FIGURE 2:**
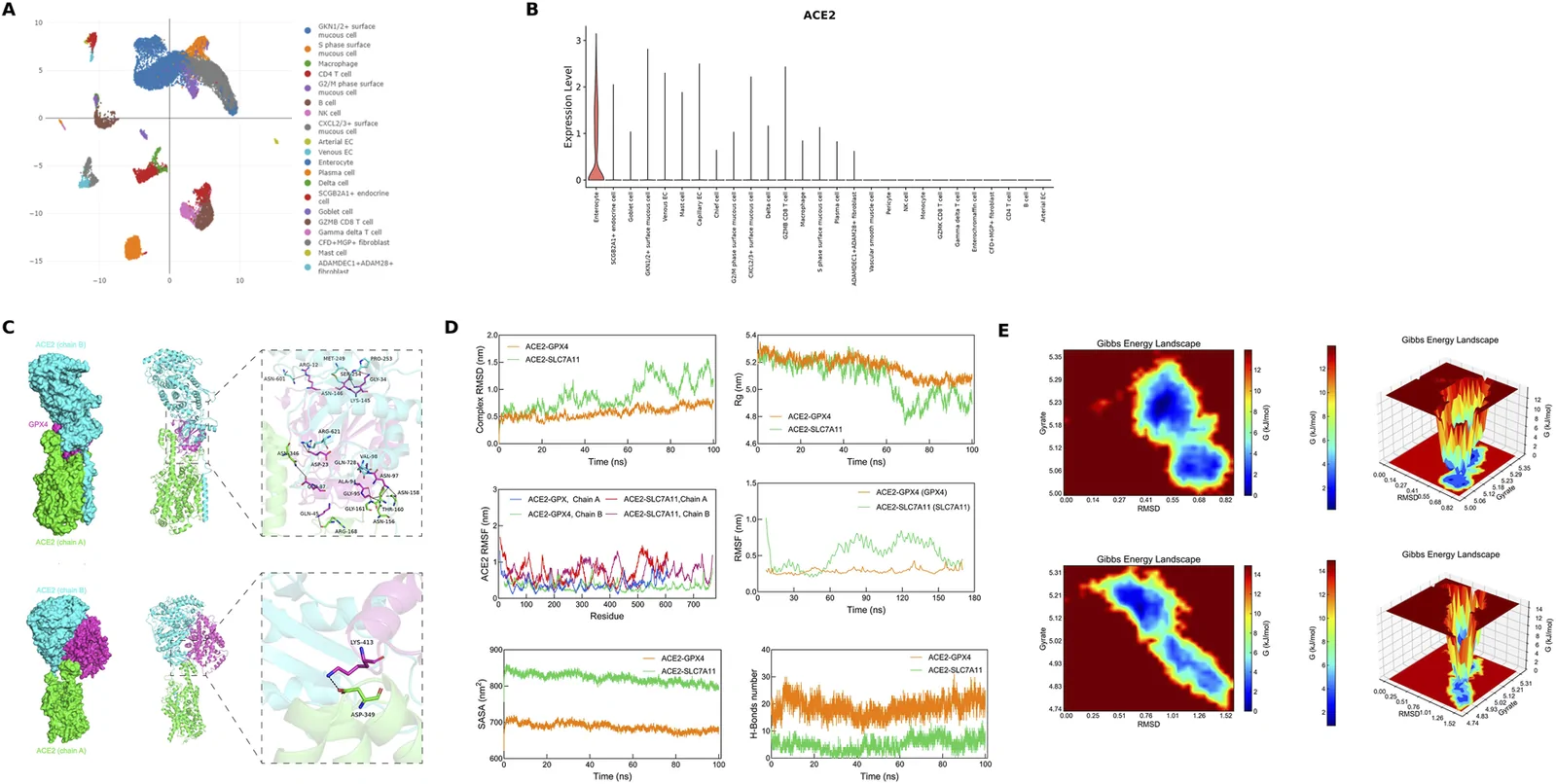
Epithelial ACE2/GPX4 interaction. **(A)** Single-cell sequencing analysis of ACE2 expression levels in the human stomach from the DISCO database. **(B)** Representative Western blot images and quantitative analysis of ACE, ACE2, and GPX4 in GEC-1 cells. GAPDH was used as an internal standard. Data are means ± SD and analyzed by Student’s t-test or one-way analysis of variance (ANOVA) with Dunnett *post hoc* test. All tests were two-sided (n = 9). P < 0.05 was considered statistically significant. *P < 0.05, versus the Ctrl group; #P < 0.05, versus the Model group. **(C)** Molecular docking (GRAMM) among ACE2, GPX4, and SLC7A11. **(D)** The RMSD, Rg, RMSF, SASA, and H-bonds number of ACE2 complexed with GPX4 and SLC7A11 during the 100 ns molecular dynamics simulation. **(E)** Gibbs free energy landscape.

As we know, the ACE2 we measured in peripheral blood is soluble ACE2 (sACE2), which is cleaved by host proteases and shed from the cell surface into the plasma (Lambert et al., 2005). We analyzed single-cell RNA-seq data from a database of DISCO and found that the ACE2 gene was distinctly expressed in mucous cells, goblet cells, enterocytes, endothelial cells, fibrocytes, B, T, and NK cells (Figure 2A). To further evaluate the expression of ACE2 in gastric epithelium, human gastric epithelial GEC-1 cells were cultured and stimulated with LPS to induce inflammation. As we expected, pretreatment with LPS led to an elevation of ACE levels, accompanied by a notable decrement in both ACE2 and GPX4 levels within GEC-1 cells (Figure 2B, P < 0.05), indicating the potential relationship of ACE2 with ferroptosis.

To elucidate whether there is a direct interaction between ACE2 and ferroptosis-related protein (SLC7A11 and GPX4), the rigid protein docking was performed with GRAMM (http://gramm.compbio.ku.edu/). As shown in Figure 2C, GPX4 is embedded between two ACE2 chains, intervening to form a stable trimer interface. Also, there is a direct interaction between ACE2 and GPX4 through hydrogen bonds. Specifically, ARG-621, GLN-45, and ASN-346 are the critical residues maintaining the protein-protein binding. However, ACE2 and SLC7A11 form hydrogen bonds with only two important residues LYS-413 and ASP-349. Subsequently, to assess the stability of the binding conformations of ACE2 and SLC7A11 or GPX4, a 100 ns molecular dynamics simulation was employed. As shown in Figure 2D, the Root Mean Square Deviation (RMSD) value shows an upward trend and fluctuates within the range of 0.5–0.8 nm (ACE2-GPX4) and 0.5–1.5 nm (ACE2-SLC7A11), respectively, while the Radius of Gyration (Rg) varies approximately between 5.0–5.2 nm (ACE2-GPX4), indicating that the compactness of the protein remains stable during the simulation. Similarly, in ACE2-GPX4, the flexible fluctuation range of chain A and chain B of ACE2 is relatively small (<1.0 nm); in ACE2-SLC7A11, the Root Mean Square Fluctuation (RMSF) of certain regions of ACE2 chain A exceeds 1 nm, indicating that ACE2 in the ACE2-SLC7A11 complex is more prone to structural changes in certain local areas and has poor stability. The Solution Accessible Surface Area (SASA) of ACE2-GPX4 stabilizes at 680–700 nm^2^, whereas ACE2-SLC7A11 has a higher SASA (860–880 nm^2^), indicating a smaller surface area exposed to the solution, possibly due to a tight binding interface and deep binding depth. To further understand the binding stability of the complex, the MM/PBSA method was employed to calculate the binding free energy using the final 20 ns stable RMSD trajectory. As shown in Figure 2E and Table 2, the ACE2-GPX4 complex is thermodynamically stable, with a ΔTotal value of −78.33 kcal/mol. These results indicated that ACE2 and GPX4 bound stably *in silico* predictions.

**TABLE 2:** MM/PBSA method calculates the average binding free energy of ACE2-GPX4 and ACE2-SLC7A11 (kcal/mol).

Energy contributions	ACE2-GPX4	ACE2-SLC7A11
ΔE_vdW_	−212.55	−198.56
ΔE_elec_	−836.36	−1296.64
ΔG_GB_	999.87	1458.89
ΔG_surf_	−29.30	−24.58
ΔG_gas_	−1048.91	−1495.20
ΔG_solvation_	970.58	1434.31
ΔG_total_	−78.33	−60.89

### 3.3 RA against LPS-induced ferroptosis via target epithelial ACE2/GPX4 interaction

**FIGURE 3:**
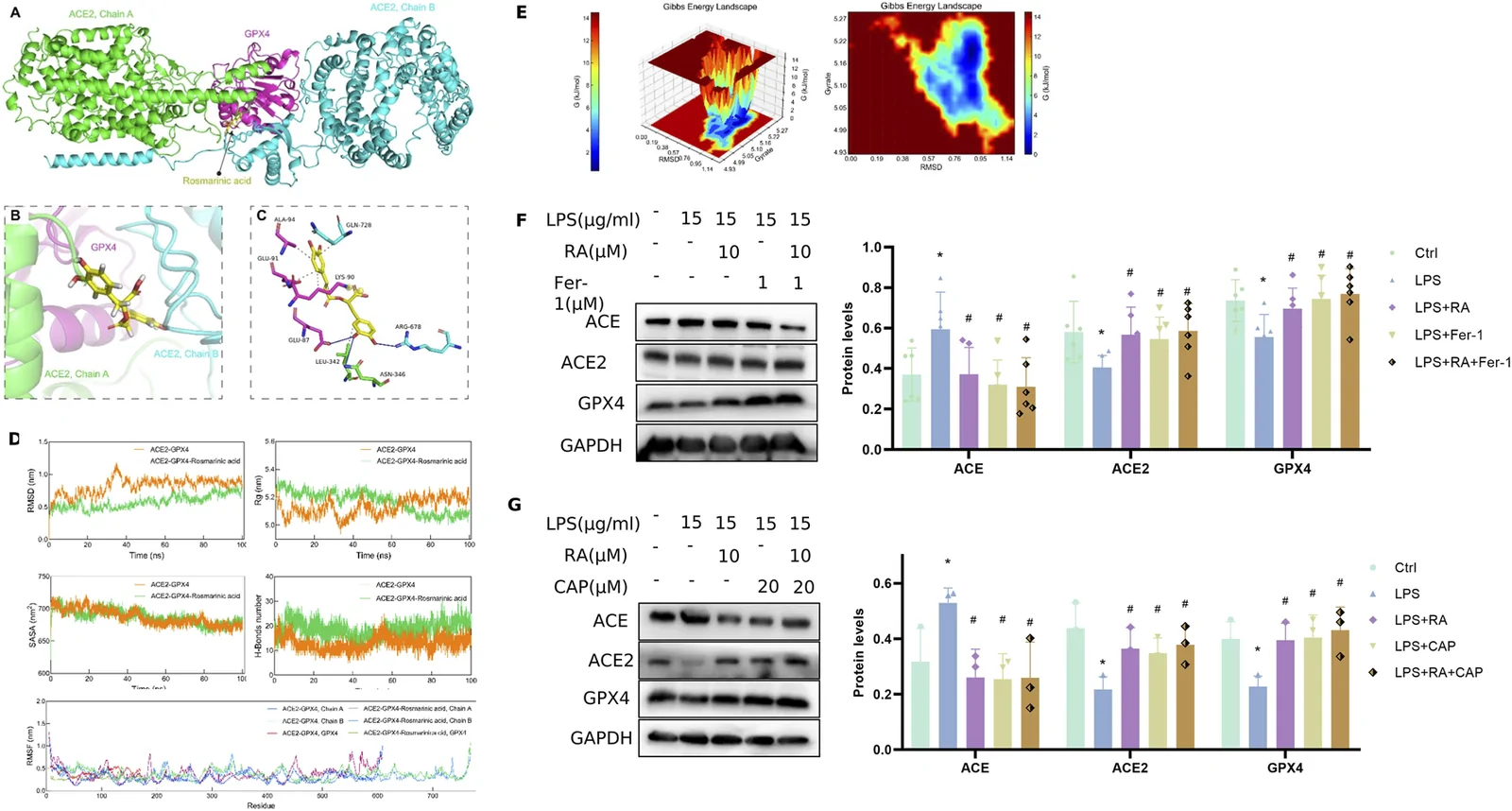
Molecular docking and molecular dynamics simulation of RA with ACE2/GPX4 interaction and *in vitro* experiment. **(A–C)** Molecular docking (AutoDock Vina v1.2.3) among RA with ACE2/GPX4 interaction. **(D)** The RMSD, Rg, RMSF, SASA, and H-bonds number of RA with ACE2/GPX4 interaction during the 100 ns molecular dynamics simulation. **(E)** Gibbs free energy landscape. **(F)** Representative Western blot images and quantitative analysis of ACE, ACE2, and GPX4 in Fer-1-treated GEC-1 cells. **(G)** Representative Western blot images and quantitative analysis of ACE, ACE2, and GPX4 in CAP-treated GEC-1 cells. GAPDH was used as an internal standard. Data are means ± SD and analyzed by Student’s t-test or one-way analysis of variance (ANOVA) with Dunnett *post hoc* test. All tests were two-sided (n = 9). P < 0.05 was considered statistically significant. *P < 0.05, versus the Ctrl group; #P < 0.05, versus the Model group.

Our previous study suggested that RA has anti-ferroptosis effects in an RAS-dependent manner (Zeng et al., 2024). Now, to reveal whether RA directly interacts with ACE2/GPX4 interactions, we first employed the AutoDock Vina v1.2.3 software to conduct a molecular docking analysis of RA with ACE2/GPX4. Our results show that the binding energy between RA and ACE2/GPX4 is −7.979 kcal/mol, indicating that RA has a strong binding affinity with ACE2/GPX4. As shown in Figures 3A–C, RA produces multiple interactions through its dual binding interface with GPX4 and ACE2, leading to a stable binding conformation. Notably, the key functional residues include GLU87, GLU91, and ALA94 of GPX4, as well as ARG678, GLN728, LEU342, and ASN346 of ACE2. A 100 ns molecular dynamics simulation was also employed. As shown in Figure 3D, the RMSD of the ACE2-GPX4-RA is stable within the range of 0.3–0.6 nm, significantly lower than ACE2-GPX4, indicating that RA significantly enhances the overall structural stability of the complex. Also, the Rg of ACE2-GPX4-RA is slightly higher than that of ACE2-GPX4, but with smaller fluctuations, maintained within 5.15–5.25 nm. The SASA of ACE2-GPX4-RA is slightly lower (approaching 660–690 nm^2^) and more compact, with a smaller exposed area. Importantly, ACE2 (Chain A, Chain B) and GPX4 exhibit reduced flexibility in multiple regions after introducing RA, such as the N-terminal, GPX4’s active site region, particularly with a noticeable decrease in GPX4’s RMSF in the 150–200 and 450–500 regions. This indicates that RA reduces the conformational flexibility of key domains and enhances local stability. The Gibbs free energy landscape shows that after the addition of RA, the system tends toward a single dominant stable state, with deeper and more concentrated conformational energy wells, and the RMSD and Gyrate distributions exhibit wider ranges (Figure 3E), indicating that the system achieves greater conformational adaptability while maintaining enhanced stability. MM/PBSA calculation results (Table 3) show that the ACE2-GPX4-RA ternary complex still exhibits significant thermodynamic stability (ΔG_total_ = −45.80 kcal/mol). The binding process is primarily driven by strong van der Waals forces (−187.93 kcal/mol) and electrostatic interactions (−803.98 kcal/mol).

**TABLE 3:** MM/PBSA method calculates the average binding free energy of RA and the ACE2-GPX4 complex (kcal/mol).

Energy contributions	ACE2-GPX4	ACE2-GPX4-rosmarinic acid
ΔE_vdW_	−212.55	−187.93
ΔE_elec_	−836.36	−803.98
ΔG_GB_	999.87	971.70
ΔG_surf_	−29.30	−25.58
ΔG_gas_	−1048.91	−991.92
ΔG_solvation_	970.58	946.12
ΔG_total_	−78.33	−45.80

To investigate whether the effects of RA on the LPS-induced gastric epithelial cell ferroptosis were dependent on epithelial ACE2/GPX4 interaction, an ACE inhibitor (Captopril, CAP) and a ferroptosis inhibitor (Ferrostatin-1, Fer-1) were utilized after LPS exposure in GEC-1 cells. Our data showed that RA significantly increased ACE2 and GPX4 expression, and decreased ACE level, regardless of the presence or absence of Fer-1 (Figure 3F, P < 0.05) or CAP (Figure 3G, P < 0.05). Moreover, there was no discernible synergistic effect between Fer-1 or CAP and RA on LPS-induced GPX4 upregulation, suggesting that the epithelial ACE2/GPX4 interaction may be the target of RA. In summary, our findings suggest that RA can prevent LPS-induced gastric epithelial cell ferroptosis via the target epithelial ACE2/GPX4 interaction.

### 3.4 RA alleviates stress-induced gastric ulcers in mice via upregulation of ACE2/GPX4 interaction

**FIGURE 4:**
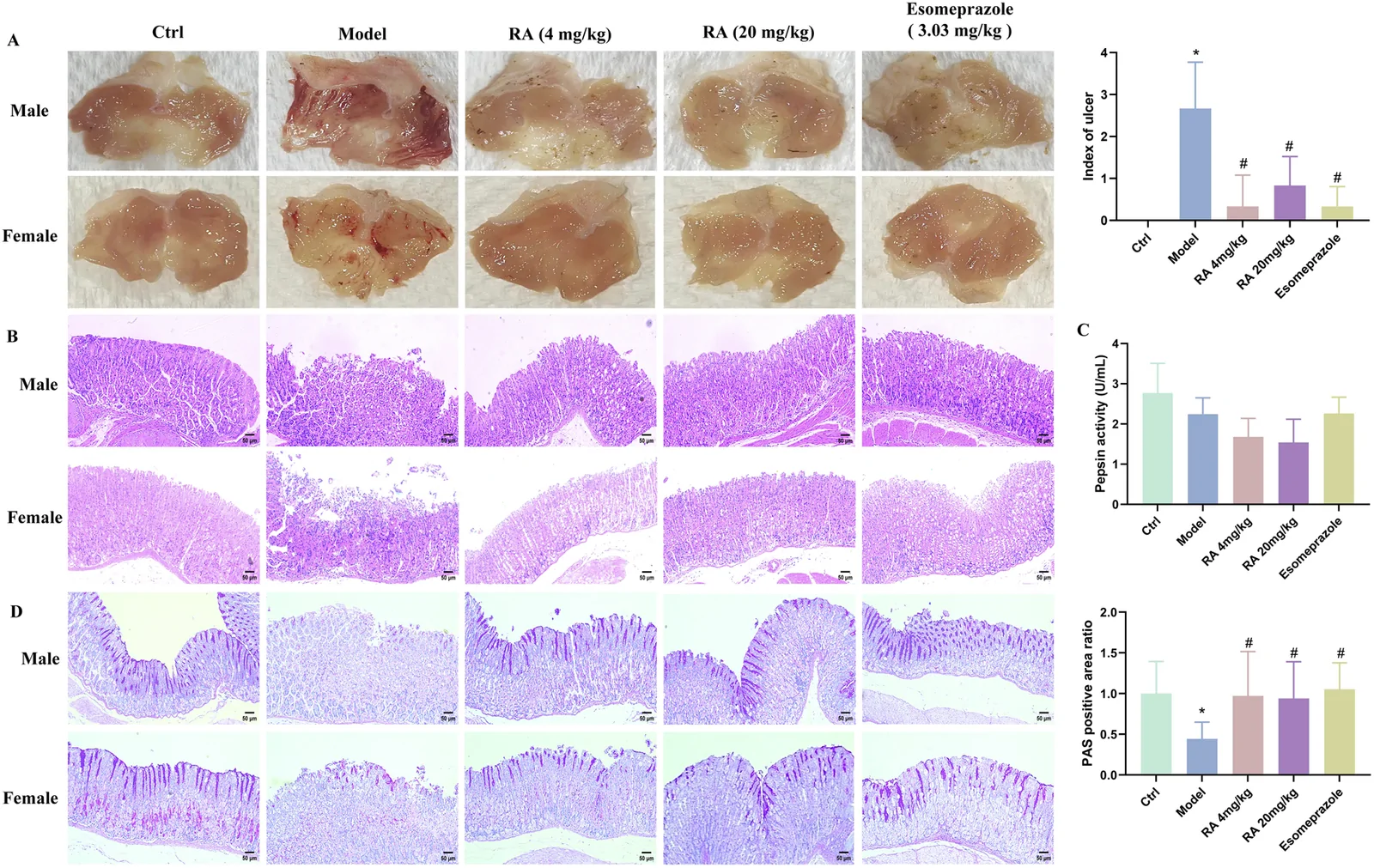
Protective effect of RA on CWIR-induced gastric ulcer in mice **(A)** Gastric tissue images of mice (left panel) and index of ulcer (right panel). **(B)** Represent HE stain in gastric tissues (scale bars: 50 μm). **(C)** Pepsin activity. **(D)** Represent PAS staining in gastric tissues (left panel, scale bars: 50 μm) and PAS-positive area ratio (right panel). Data are means ± SD and analyzed by Student’s t-test or one-way analysis of variance (ANOVA) with Dunnett *post hoc* test. All tests were two-sided (n = 3). P < 0.05 was considered statistically significant. *P < 0.05, versus the Ctrl group; #P < 0.05, versus the Model group.

To explore the preventive value of RA on stress-induced gastric ulcers, we performed *in vivo* experiments with CWIR mouse models. As shown in Figure 4A, the control group mice exhibited no significant bloating, with distinct vascular distribution on the gastric surface, intact and smooth gastric mucosa, and no signs of ulcers, bleeding, congestion, edema, or blood clots. After 6 h of cold-water immersion and restraint, the gastric mucosa partially sloughed off, with scattered bleeding points and linear ulcers. The gastric contents appeared darker, indicating substantial intragastric bleeding. RA (4 and 20 mg/kg) treatment groups demonstrated significant protective effects on the gastric mucosa, with reduced bleeding. Similarly, esomeprazole treatment decreased gastric bleeding and the ulcer index in mice (P < 0.05).

Histologically, the control group mice had intact gastric mucosa with well-aligned gastric glands and no evident hyperplasia. In contrast, the model group mice showed interrupted mucosa with ulcer formation, inflammation, and bleeding in the mucosa and submucosa. As expected, the RA treatment group exhibited relatively intact mucosa, with some ulcers still present but a more organized gland structure compared with the model group (Figure 4B). However, no statistical significance was observed in pepsin activity (Figure 4C, P > 0.05), indicating that the short-term effects of ulcers induced by CWIR on pepsin activity may be negligible.

**FIGURE 5:**
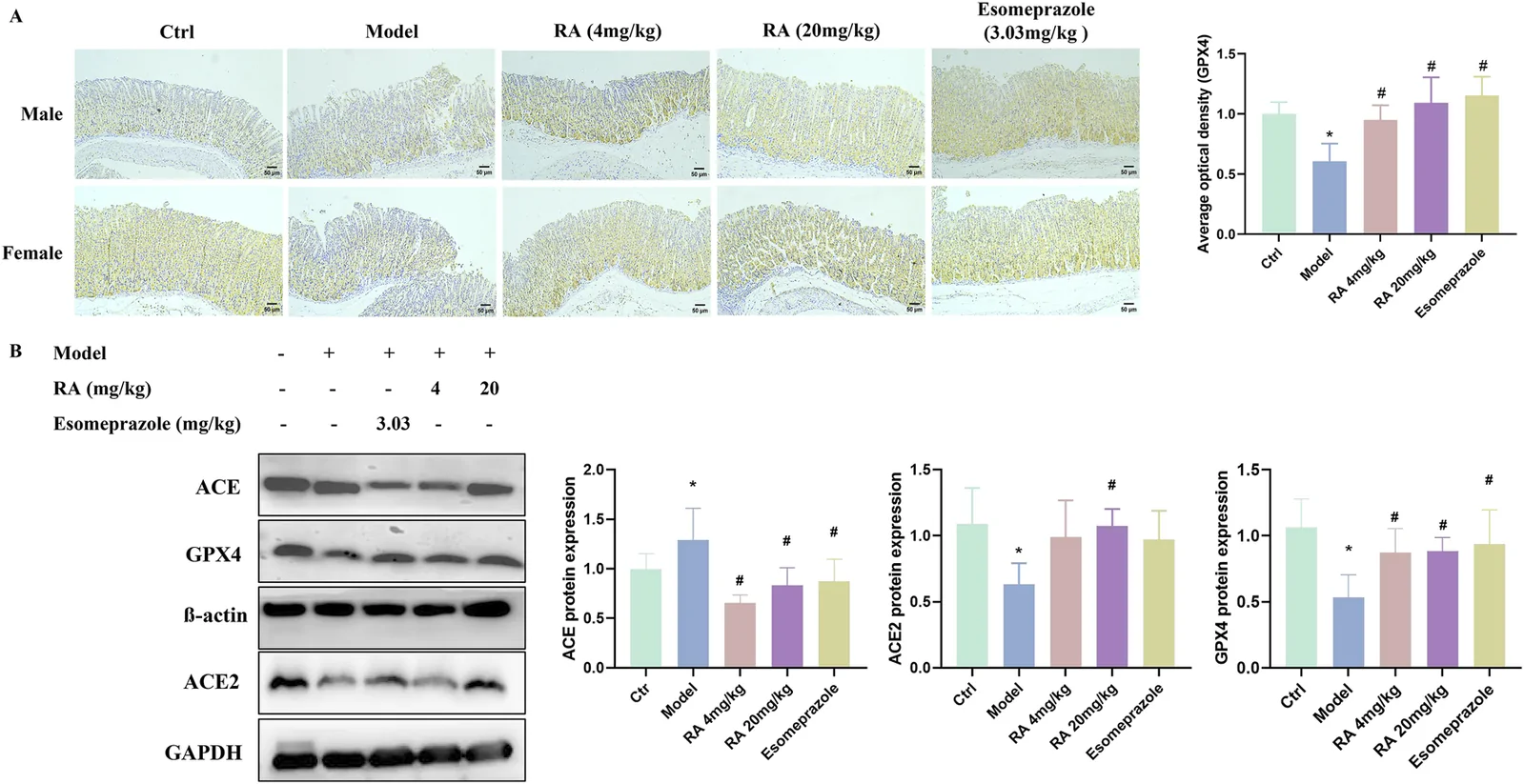
Effect of RA on RAS and GPX4 levels in CWIR-induced gastric ulcer mice **(A)** IHC staining for GPX4 expression in gastric tissues of CWIR-induced gastric ulcer mice (left panel) and the average optical density of GPX4. **(B)** Representative Western blot images and quantitative analysis of ACE, ACE2, and GPX4 in mice. Data are means ± SD and analyzed by Student’s t-test or one-way analysis of variance (ANOVA) with Dunnett *post hoc* test. All tests were two-sided (n = 4). P < 0.05 was considered statistically significant. *P < 0.05, versus the Ctrl group; #P < 0.05, versus the Model group.

To assess whether RA protects against CWIR-induced gastric damage, we performed PAS staining of gastric tissues. As shown in Figure 4D, the control group exhibited normal surface and neck epithelial cells within the gastric pit, accompanied by a strong PAS stain. The PAS staining of the mucus in the stomach from the model mice group was weaker than that in the control group, which was notably increased by RA and esomeprazole treatment (P < 0.05). Importantly, our GPX4 IHC data showed that the anti-ferroptosis protein GPX4 level was significantly decreased in the model mice (Figure 5A, P < 0.05). RA could increase GPX4 expression as well as esomeprazole (Figure 5A, P < 0.05), which was consistent with the western blot result (Figure 5B, P < 0.05). Our western blot data further demonstrated that CWIR significantly increased the ACE level while decreasing the ACE2 level in the stomach, whereas RA treatment could partially restore both these levels (Figure 5B, P < 0.05). Taken together, these data suggest that RA alleviates CWIR-induced gastric ulcers in mice via regulation of ACE2/GPX4.

## 4 Discussion

In the current study, our RNA-seq data and bioinformatics analysis revealed that ferroptosis and ACE2 might contribute to the occurrence of gastric ulcers. Furthermore, the epithelial ACE2/GPX4 interaction might be a key mechanism of gastric ulcers. Simultaneously, our in-silico predictions revealed a stable binding interaction between RA and the ACE2/GPX4 interaction, which may provide a pivotal target for SGU. Finally, our *in vivo* data suggest that RA alleviates CWIR-induced gastric ulcers in mice via regulation of the ACE2/GPX4 interaction.

Initially, we collected biopsy samples of gastric mucosa from patients and identified 14 DEGs related to ferroptosis between cases of chronic gastritis and gastric ulcer. Notably, PROK2, DUOX1, and TIMP1 establish a PPI network with RAS-associated proteins (ACE and ACE2). ACE2, the central effector molecule of the RAS system as a homolog of ACE, is widely expressed in the kidney, heart, brain, eye, lung, liver, pancreas, and gastrointestinal tract (Gheblawi et al., 2020). Especially in the gastrointestinal tract, ACE2 plays a vital role in maintaining the integrity of the intestinal epithelial barrier and regulating intestinal glucose transport (Duan et al., 2019; Visvanathan et al., 2025). Tissue ACE2 (cACE2) is a transmembrane protein, which ectodomain is cleaved by host proteases and shed from the cell surface into plasma as the soluble form of ACE2 (sACE2) (Lambert et al., 2005). Accumulating evidence has indicated that elevated levels of circulating sACE2 were correlated with diverse inflammatory diseases, such as cardiovascular disease, diabetes mellitus, recurrent stroke, and inflammatory bowel disease (Xue et al., 2025; Shahrokh et al., 2024). Consistent with these, our ELISA analysis demonstrated the significantly increased serum ACE2 concentration in gastric ulcer patients, suggesting that gastric ulcers may enhance sACE2 release. Moreover, in GEC-1 cells and CWIR mice, cACE2 was significantly downregulated.

The desquamation of surface epithelial cells serves as the definitive indicator of gastric mucosa damage. In this study, CWIR mice exhibited significant desquamation of epithelial cells, accompanied by downregulation of GPX4. GPX4 serves as a pivotal repressor of ferroptosis, with its activity contingent upon the glutathione (GSH) produced through the activation of the cystine-glutamate antiporter SLC7A11 (Yang et al., 2014). Our prior research has shown that GPX4 is regulated by ACE/ACE2 in bronchial epithelial cells (Zeng et al., 2024). In this study, we get similar results. Importantly, molecular docking results demonstrated that ACE2 was easy to interact with GPX4 directly, but not with SCL7A11. The subsequent findings obtained through molecular dynamics simulation further furnished compelling evidence that the ACE2-GPX4 complex demonstrates enhanced structural stability, reduced flexibility fluctuations, and augmented hydrogen bonding network stability, hinting that the epithelial ACE2/GPX4 interaction may constitute a pivotal mechanism underlying gastric ulcer pathogenesis.

Our previous study reported that RA has a similar binding energy with both ACE and ACE2, implying the multiple-target role of RA (Zeng et al., 2024). In the present study, we employed the AutoDoc Vina v1.2.3 software to conduct a molecular docking analysis of RA with ACE2/GPX4. As we expected, RA was easy to interact with ACE2/GPX4. The molecular dynamics simulation results also indicated that RA can further enhance the stability of the interaction between ACE2 and GPX4, suggesting ACE2/GPX4 potential as a therapeutic target of RA for SGU intervention. To further elucidate whether ACE2/GPX4 is the potential target of RA, we employed two inhibitors (CAP and Fer-1) to suppress ACE and ferroptosis, respectively. It is noteworthy that neither of them can enhance the impact of RA on upregulating ACE2 and GPX4 further. Therefore, epithelial ACE2/GPX4 interaction may be the target of RA. Furthermore, *in vivo* experiments demonstrated that the impact of RA on mitigating SGU was comparable to that of Esomeprazole, the S-isomer of omeprazole with lower first-pass metabolism and higher bioavailability (Vachhani et al., 2009).

In summary, this study predicted that ACE2 might form a stable protein complex with GPX4 *in silico*. Furthermore, the epithelial ACE2/GPX4 interaction might be a key target for treating gastric ulcers. Moreover, RA alleviates stress-induced gastric ulcers in mice via upregulation of the ACE2/GPX4 interaction. These findings provide new insight into the pathogenesis of SGU and offer scientific support for developing potential therapeutic interventions targeting the ACE2/GPX4 interaction in SGU.

However, there are some limitations in the present study. First, *in silico* simulation results only provide predictive evidence rather than definitive proof of physical binding between ACE2 and GPX4. In the future, we intend to design Co-IP, PLA, or alternative protein interaction verification assays to furnish evidence of direct interaction between the ACE2/GPX4 complex. Second, it must be further examined whether RA directly targets this complex. Future studies may consider employing surface plasmon resonance technology to conduct drug-protein binding experiments, and ACE2/GPX4 knockdown and overexpression rescue experiments to directly verify whether RA exerts its protective effect on gastric mucosa by specifically targeting the ACE2/GPX4. Third, some key functional indicators of ferroptosis, such as lipid peroxides (MDA, 4-HNE), iron content, and the GSH/GSSG ratio, were not measured. In the future, we will systematically assess the anti-ferroptosis role of RA *in vivo* and *in vitro*.

## Data Availability

The original contributions presented in the study are publicly available. These data can be found here: China National Center for Bioinformation, accession number: PRJCA071865.
